# SIEVEseq: unified differential expression, variability, and skewness analyses using RNA-Seq data

**DOI:** 10.1093/dnares/dsag012

**Published:** 2026-07-29

**Authors:** Hongxiang Li, Tsung Fei Khang

**Affiliations:** School of Mathematics, Yunnan Normal University, Kunming, Yunnan 650500, P.R. China; Yunnan Key Laboratory of Modern Analytical Mathematics and Applications, Yunnan Normal University, Kunming, Yunnan 650500, P.R. China; Institute of Mathematical Sciences, Faculty of Science, Universiti Malaya, Kuala Lumpur 50603, Malaysia; Institute of Mathematical Sciences, Faculty of Science, Universiti Malaya, Kuala Lumpur 50603, Malaysia; Universiti Malaya Centre for Data Analytics, Universiti Malaya, Kuala Lumpur 50603, Malaysia

**Keywords:** compositional data analysis, differential expression analysis, differential variability and skewness, RNA-Seq, skew-normal distribution

## Abstract

RNA-Seq data analysis is commonly biased towards detecting differentially expressed genes and insufficiently conveys the complexity of gene expression changes between biological conditions. This bias arises because discrete count models cannot fully and independently parameterize the mean, variance, and skewness of gene expression distributions. Therefore, a unified statistical framework that simultaneously tests differential expression, variability, and skewness is needed. We present SIEVEseq, a statistical methodology that provides such a framework. SIEVEseq embraces a compositional data analysis strategy to transform discrete RNA-Seq counts into continuous form with a distribution well-fitted by the skew-normal distribution. Both parametric and nonparametric simulations show that SIEVEseq better controls the false discovery rate and Type II error than existing differential expression methods. Analysis of the Mayo RNA-Seq dataset for Alzheimer's disease demonstrates that gene sets with significant differences in mean, variance, and skewness between control and disease groups strongly predict disease state. Furthermore, functional enrichment analysis indicates that relying solely on differentially expressed genes identifies only part of the biological spectrum, whereas incorporating genes with differential variability and skewness reveals additional disease-related aspects. Cross-data and cross-methodology validation suggest the detected biological signals are genuine. The SIEVEseq R package is available at https://cran.r-project.org/web/packages/SIEVEseq.

## 1. Introduction

The goal of gene expression analysis is to uncover genes with patterns of expression variation that are significantly associated with variation of some biological states. Three such patterns are of potential biological interest: (i) differential expression (DE), (ii) differential variability (DV), and (iii) differential skewness (DS). These correspond to shifts in the mean, variance, and skewness of gene expression levels, respectively, when comparing 2 biological conditions.

The statistical detection of genes that are significantly up- or down-regulated (ie DE genes) forms the main use of RNA-Seq data, but not all genes that are associated with change in biological states show change in mean expression level. For example, a gene's previously tightly controlled expression range can be lost, resulting in expression over a much wider range.^1,2^ Genes associated with modulation of the immune system, stress, and hormonal regulation have high gene expression variability.^3^ Furthermore, gene expression variability often shows consistent differences between individuals with and without certain disease states.^4–6^ In cancer biology, cancer tissues show higher variability of gene expression compared with normal tissues.^6,7^ In all these cases, the focus is on genes that show DV (ie DV genes). DV describes changes in the spread of expression values across samples and is often interpreted as a gain or loss of regulatory control, as well as increased or decreased inter-individual heterogeneity.^4^ Unfortunately, current methods of detecting DV genes are poorly integrated with the framework of commonly used DE tests. Differences in the skewness of gene expression levels have also been suggested to affect disease pathogenesis.^8–11^ Skewness characterizes the shape of the distribution of gene expression levels and potentially captures more nuanced patterns of variation compared with the mean and the variance. DS describes differences in the asymmetry of expression distributions in 2 groups, particularly those induced by presence of nontrivial extreme expression levels. Church et al.^10^ reported elevated positive skewness in the expression of genes within immune-related pathways when comparing cancer against control subjects. No methods are currently available for detecting the class of genes that show DS.

Conventional discrete count models have been effective in developing useful statistical tests of DE, but the lack of model parameters that explicitly characterize variance or skewness makes the development of DV and DS tests difficult. For context, we first provide an overview of DE and DV tests in RNA-Seq data analysis. The discrete negative binomial (NB) model underlies popular DE methods such as edgeR^12,13^ and DESeq2.^14^ For the NB model, the variance $\sigma^{2}$ is modelled as a linear ($\sigma^{2}=\phi \mu$) or quadratic ($\sigma^{2}=\mu +\phi \mu^{2}$) function of the population mean *μ*, with *ϕ* as the dispersion parameter. These 2 parametrizations lead to the NB1 and NB2 models, respectively. However, since mean and variance are confounded, it may be difficult to use these models effectively for analysis of gene expression variability.^15^ Other models such as the generalized Poisson^16^ and the Poisson–Tweedie distribution^17^ have also been used. Alternatively, the voom algorithm^18^ assumes a Gaussian distribution for the logarithm of RNA-Seq counts, and repurposes the limma pipeline^19^ used in microarrays for DE analysis. Gauthier et al.^20^ proposed dearseq, a distribution-agnostic method that uses a test statistic based on the variance component score. Nonparametric methods (NOISeq^21,22^ and SAMSeq^23^) are available, but they are less commonly used compared to DESeq2 or edgeR. Nevertheless, for large sample sizes, the Wilcoxon rank-sum test seems superior to edgeR, DESeq2 and dearseq with respect to the false discovery rate (FDR).^24^

In contrast, only 5 DV methods are available: (i) DiffVar,^25^ an empirical Bayes method that uses the limma framework;^19^ (ii) MDSeq,^26^ which uses an NB1 generalized linear model to test DE and DV separately; (iii) the generalized additive model for location, scale and shape,^27^ which tests the effects of biological factors on a gene’s Poisson and non-Poisson variation using the NB2 model;^28^ (iv) DiffDist,^29^ a hierarchical Bayesian model based on the NB2 distribution; and (v) clrDV,^30^ our contribution that uses the compositional data framework in the present work but restricted to DV test.

The non-normality of gene expression distribution, particularly as characterized by skewness, may be potentially crucial under certain biological contexts.^11^ It can shed light on gene functionalities that cannot be discovered using conventional analysis of differences in mean—and, less commonly, differences in variability.^4,5,31^ The relevance of skewness of distributions of gene expression as measured using microarray data was documented in several studies.^8,9,32,33^ More recently, Church et al.^10^ highlighted skewness as an effective indicator of biological heterogeneity in gene pathways associated with specific cancer cohorts.^10^ Despite these studies, no methods are currently available for testing DS.

A unified framework that enables the simultaneous testing of mean, variance and skewness requires a model where these parameters are explicit and independent of one another. This suggests transformation of RNA-Seq count data into continuous data, followed by their appropriate modelling using a continuous distribution. Quinn et al. adopted a compositional data framework for RNA-Seq data and repurposed ALDEx2^34,35^—originally developed for differential abundance testing in microbiome data—for detecting DE genes.^36^ ALDEx2 incorporates a Dirichlet process to produce centred log-ratio (CLR) transformation of count data.^37^ The transformed data is assumed Gaussian, and a Welch *t*-test or Wilcoxon test is applied to test for differential abundance between 2 populations. Interestingly, ALDEx2 has been shown to be competitive against edgeR and DESeq2.^36^ Although the Welch *t*-test is robust against non-Gaussian data,^38^ without explicitly modelling the CLR-transformed data, it is unclear how tests concerning aspects such as equality of variances or skewness can be done.

Towards the goal of a unified framework for RNA-Seq data analysis, we need to use a model that is both mathematically tractable and provides a good empirical fit to the transformed RNA-Seq data. Our solution is SIEVEseq (unified DE, variability, and skewness analyses using RNA-Seq data), which can perform simultaneous testing of DE, variability, and skewness of genes using RNA-Seq data. SIEVEseq adopts a compositional data analysis approach to modelling discrete RNA-Seq count data, applies Aitchison's CLR transformation^37^ to convert them into continuous form, and uses the skew-normal distribution^39^ to model them. With SIEVEseq, genes with significant variation in skewness can now be detected for the first time, alongside those with significant variation in mean and variance.

## 2. Materials and methods

### 2.1. The compositional data framework and the centred log-ratio transformation

Let $X_{gi}$ be the read count of gene *g* and sample *i*, where g=1,2,…,G and i=1,2,…,n. The *i*th sample data become compositional if we normalize the counts by dividing them by the sum of gene counts. Application of the CLR transformation converts the variable space from the simplex to the Euclidean space, for which standard mathematical and statistical methods operate. The CLR transformation of the count of the *g*th gene is defined as the logarithm of the gene count divided by the geometric mean of gene counts across all *G* genes. To avoid multiplication or division by 0, a pseudo-value of 0.5 replaces any zero gene counts.

### 2.2. Modelling centred log-ratio-transformed data using the skew-normal distribution

Denote the CLR-transformed count from gene *g* in sample *i* by $Y_{\mathrm{gi}}$. We model the random variable $Y_{\mathrm{gi}}$ using a skew-normal distribution with centred parameters. Notationally, we write $Y_{gi}\;∼\;SN_{C}(\mu_{g},\sigma_{g},\gamma_{g}),$ where $\mu_{g}$ is the mean, $\sigma_{g}$ is the standard deviation, and $\gamma_{g}$ is the skewness parameter (see Materials S1 for mathematical details). The parameter vector $\theta_{g}^{\left(C\right)}\;=\;(\mu_{g},\sigma_{g},\gamma_{g})$ has parameter space $R\times R^{+}\times (-k,k)$, where $k\;=\;\sqrt{2}(4-\pi )/(\pi -2{)}^{3/2}\;\approx \;0.9953$. The skew-normal distribution simplifies to the normal distribution with mean $\mu_{g}$ and variance $\sigma_{g}^{2}$ when $\gamma_{g}\;=\;0$. Given 2 populations and sufficiently large sample sizes, tests of equality of mean, standard deviation and skewness can be done using the Wald statistic:


$$Z_{g\;}=\;\frac{{\hat{\theta }}_{g,2}-{\hat{\theta }}_{g,1}}{\sqrt{\mathrm{Var}({\hat{\theta }}_{g,2})+\mathrm{Var}({\hat{\theta }}_{g,1})}},$$

where g=1,2,…,G index the genes and j=1,2 index the groups. Denote the maximum likelihood estimator or the maximum penalized likelihood estimator^40,41^ as ${\hat{\theta }}_{g,j}$. Then, for testing DE, ${\hat{\theta }}_{g,j}\;=\;{\hat{\mu }}_{g,j}$; for testing DV, ${\hat{\theta }}_{g,j\;}=\;{\hat{\sigma }}_{g,j}$; and for testing DS, ${\hat{\theta }}_{g,j}\;=\;{\hat{\gamma }}_{g,j}$. For estimating $\mathrm{Var}({\hat{\theta }}_{g,j})$, the inverse of the Fisher information matrix of $\theta_{g,j}^{\left(C\right)}$ is used. When sample size is sufficiently large, $Z_{g}$ converges to the standard normal distribution. The Benjamini–Yekutieli (BY) procedure,^42^ which allows for arbitrary dependence between the tested hypotheses, is used to control the FDR and adjust *p*-values for multiple hypothesis testing.

Under the SIEVEseq framework, 8 gene classes are possible (see also Fig. S1). These range from genes that do not show DE, DV or DS, to genes that simultaneously show them (Table 1).

**Table 1.** Eight gene classes identifiable using SIEVEseq.

Gene class	DE	DV	DS	Label
Non-DE, non-DV and Non-DS	0	0	0	000
Pure DE	1	0	0	100
Pure DV	0	1	0	010
Pure DS	0	0	1	001
DE, DV, non-DS (DEV)	1	1	0	110
DE, non-DV, DS (DES)	1	0	1	101
Non-DE, DV, DS (DVS)	0	1	1	011
DE, DV, DS (DEVS)	1	1	1	111

1 = true, 0 = false.

### 2.3. Data description and preprocessing

To assess the suitability of the skew-normal distribution for fitting CLR-transformed counts and to compare SIEVEseq's performance with existing methods for DE tests, with a particular focus on controlling the FDR and the probability of Type II error, it is essential to simulate RNA-Seq count data distributions using both realistic parameter values and a nonparametric resampling strategy. For this purpose, we used 3 real RNA-Seq datasets with varying sample sizes to facilitate reliable parameter estimation of the NB2 model. Two datasets with sufficiently large sample sizes in each group were used to enable a nonparametric resampling–based simulation design.

The first dataset (GEO accession number: GSE123658) contains whole blood RNA-Seq data from 39 Type 1 diabetes patients and 43 healthy donors, with 16,785 genes.^43^ The second dataset (GEO accession number: GSE150318) consists of longitudinal RNA-Seq data from 114 short-lived killifish *Nothobranchius furzeri*,^44^ with samples collected at 10 and 20 wk of age, covering 26,739 genes. The third dataset (GEO accession number: GSE179250) comprises 29,245 genes from 192 human liver samples.^45^ The fourth dataset (GEO accession number: GSE229705) comprises RNA-Seq profiles of 60,591 genes across 123 paired tumour and normal samples.^46^ The fifth dataset (GEO accession number: GSE150910) consists of RNA-Seq profiles of 18,838 genes measured in whole-lung tissues from 82 chronic hypersensitivity pneumonitis patients, 103 idiopathic pulmonary fibrosis (IPF) patients, and 103 unaffected controls.^47^ These datasets will subsequently be referred to as the Valentim, Kelmer, Zhou, Dolgalev, and Furusawa datasets, respectively.

For empirical assessment, we used the Mayo RNA-Seq dataset,^48^ which contains 278 samples and 64,253 transcripts. Access to this dataset was obtained from the AD Knowledge Portal (https://adknowledgeportal.synapse.org) after obtaining permission from the database owner (1 September 2022; ID: 9603055). This dataset comprises RNA isolated from the temporal cortex of patients with 4 biological conditions: control (n=80), Alzheimer's disease (AD; n=84), progressive supranuclear palsy (n=80), and pathologic ageing (n=30). Here, we compared the AD group against the control group. We chose the Mayo RNA-Seq dataset for 3 main reasons. Firstly, the study has a rigorous experimental design and quality control for ensuring data fidelity. Secondly, the sample size per group is sufficiently large (50 or more per group) to allow the standard deviation and skewness parameters of the skew-normal model to be reliably estimated.^40,49^ Thirdly, analysis of an AD dataset enables us to leverage on substantial literature to cross-check the biological significance of genes detected using SIEVEseq.

For cross-data validation of gene ontology (GO) biological processes identified from the Mayo RNA-Seq dataset, we used the Nakayama dataset^50^ (GEO accession number: GSE249477) as an external cohort. This dataset comprises whole blood RNA-Seq profiles of 21,479 genes from a cohort of 62 subjects from Japan. The study participants were clinically categorized into 3 groups: healthy controls (n=21), patients with mild cognitive impairment (n=20) due to AD, and patients with AD (n=21). We focused on the 21 AD patients and 21 controls to provide a direct comparison with the primary Mayo cohort.

To filter genes, those with an average counts per million (CPM) ≤0.5 or with zero counts in at least 85% of the samples were excluded. After this step, the Valentim, Kelmer, Zhou, Dolgalev, and Furusawa datasets retained 12,283, 16,670, 17,862, 20,151, and 14,316 genes, respectively. In the Mayo RNA-Seq dataset, after removing samples with missing class labels, 78 and 82 samples remained for the control and AD groups, respectively. Similarly, in the Nakayama dataset, 13,078 genes remained for the AD-control comparison after handling missing values and gene filtering.

For SIEVEseq and ALDEx2, the CLR transformation was applied to the raw counts. For the 8 DE methods (edgeR, DESeq2, voom, tweeDEseq, NOISeq, DSS, dearseq, and Wilcoxon rank-sum test), raw counts were normalized using the trimmed mean of M values approach.^51^

### 2.4. Parametric simulation design for evaluating performance of DE tests

To compare the performance of SIEVEseq with existing DE methods, RNA-Seq count data were simulated from an NB2 model with model parameters estimated from real datasets using polyester^52^ R. Three RNA-Seq datasets with reasonably large sample sizes were used to facilitate reliable estimation of the standard deviation and skewness parameters.

Assuming the distribution of the RNA-Seq count data follows the NB2 model, we randomly sampled 5000 of the filtered genes and then estimated the parameters of the NB2 model using samples from one group (Type 1 diabetes group in the Valentim dataset (n=39), 10-wk group in the Kelmer dataset (n=114), all liver samples in the Zhou dataset (n=192)). After this, we generated 200 biological replicates from the estimated NB2 models. The goodness-of-fit of the skew-normal model on the distribution of the CLR-transformed simulated count data was checked using the Kolmogorov–Smirnov (KS) test.

Using the FDR and the probability of Type II error (β) as performance metrics (see Materials S1.5), we compared SIEVEseq against 9 other DE methods: edgeR, DESeq2, voom, DSS, tweeDEseq, NOISeq, ALDEx2, dearseq, and the Wilcoxon rank-sum test. We chose edgeR, DESeq2 and voom because of their popularity as DE tests. Then, to comprehensively survey the landscape of DE tests using a diversity of approaches, we included 2 nonparametric methods: NOISeq and the Wilcoxon rank-sum test, as well as DSS, dearseq and tweeDEseq, which are based on ideas of dispersion shrinkage, variance component score, and the Poisson–Tweedie model, respectively. RNA-Seq data were similarly simulated using the same 3 datasets with polyester^50^ R. For each of the 3 datasets, a total of 2,000 filtered genes were randomly selected, and their mean (μ) and size (1/ϕ) parameters under assumption of an NB2 model were estimated. The samples used were the 39 Type I diabetes patients for the Valentim dataset, the 114 fish at 10 wk of age for the Kelmer dataset, and all 192 livers for the Zhou dataset.

In a 2-group comparison setting, both groups have identical NB2 model parameters under the null hypothesis of equal mean. Hence, to simulate DE genes, we spiked 10% of the genes in 1 of the 2 groups to be differentially expressed, approximately half of which are up-regulated and the remainder down-regulated. This was done by multiplying or dividing their estimated mean parameter by a random value uniformly chosen between 2 and 4. Biological replicates of size 30, 50, and 100 per group were generated from the NB2 model, with 30 instances simulated for all 3 datasets.

We defined the region of desirable performance as where both FDR and *β* are below 0.05. Then, we computed the percentage of simulated instances falling within this region (θ). We identified genes with adjusted *P*-value below 0.05 as DE genes, and recorded the computing time of each method.

To evaluate performance similarity of DE methods, we used a cluster heatmap with the Ward clustering algorithm and Euclidean distance for between-method distances. We computed the mean and standard deviation of FDR and *β* (used as features) for the 30 simulated instances across each of the 3 sample size scenarios, and subjected them to min-max normalization before constructing the cluster heatmap.

Finally, to evaluate the effect of using pseudo-values that are smaller than 0.5, we conducted a sensitivity analysis using 2 smaller pseudo-values (0.01 and 0.1).

### 2.5. Nonparametric simulation design for evaluating performance of DE tests

To further validate whether the superior performance of SIEVEseq persists when the underlying distribution deviates from the NB assumption, we implemented a nonparametric simulation study using the SimSeq^53^ R package. This approach constructs simulated counts by resampling from the empirical distributions of a real dataset, and captures the empirical variance and skewness inherent in biological samples. To obtain moderately large sample sizes in the simulated conditions, we used 2 RNA-Seq datasets (Dolgalev dataset: 123 paired tumour-normal samples; Furusawa dataset: 103 IPF patients vs. 103 controls) with sufficiently large sample sizes in each group.

We randomly selected 2,000 filtered genes for each simulation pool. Following the SimSeq protocol, 10% of these genes were spiked to become DE genes with an absolute log fold-change >1. Under this nonparametric framework, biological replicates were generated for 2 sample sizes (30 and 50 per group), with 30 independent instances simulated for each scenario. We restricted the maximum sample size to 50 to satisfy the SimSeq operation constraint, $2n\leq \max\{N_{1},N_{2}\}$, where $N_{1}$ and $N_{2}$ are the sample sizes in the 2 respective groups of the source dataset.

Consistent with the design of our parametric simulation evaluation, we also used FDR and *β* to assess model performance. We compared SIEVEseq against 8 other DE methods: edgeR, DESeq2, voom, DSS, tweeDEseq, NOISeq, ALDEx2, and dearseq. The Wilcoxon rank-sum test was excluded to avoid circular bias, as the SimSeq algorithm itself applies this test to define the ground-truth DE genes. We defined the region (*θ*) of desirable performance where FDR <0.05 and β<0.2, and subsequently computed the percentage of simulated instances falling within this region. Genes identified as DE genes with an adjusted *P*-value below 0.05. Finally, the similarity of DE methods was evaluated using the same cluster heatmap analysis and hierarchical clustering parameters as described in the parametric simulation study.

### 2.6. Empirical validation

We applied SIEVEseq to the Mayo RNA-Seq dataset to assess its capability for detecting DE, DV, and DS genes that are contextually meaningful in AD. The KS test was used to check the fit of the skew-normal distribution on the CLR-transformed count data.

For comparison of DE methods, we selected those that showed reasonably good performance in the simulation study. Comparison of DV methods was reported previously by us.^30^ Volcano plots were used to inspect joint patterns of biological and statistical significance. Biological significance was measured using the difference of mean for DE genes, $\mathrm{lo}g_{2}$ of the ratio of standard deviation for DV genes, and the difference of skewness for DS genes. Thus, zero corresponds to genes with no significant biological difference between the 2 groups. Significant DE, DV and DS genes were flagged using the significance score method (see Materials S2).^54^ Sets of DE genes detected using the different methods were examined using Venn diagrams. Finally, the joint distributions of estimated mean, standard deviation, and skewness parameters for the control and the AD group were visualized using contour heatmaps.

Subsets of the genes detected using SIEVEseq that are strongly predictive of the AD state were identified using the Generalized, Unbiased Interaction Detection and Estimation (GUIDE Version 40.3) decision tree algorithm.^55,56^ To assess the informativeness of the set of significant DE, DV, and DS genes (the top 10 genes on the left and the right tails of the distribution of biological significance scores) for predicting the AD state, we estimated the out-of-bag generalization error using the GUIDE random forests model with default hyperparameters (equal priors, unit misclassification costs, univariate split highest priority, no interaction splits, number of trees = 1,000, SE parameter = 0.25). For benchmarking, AD state classification using GUIDE was done using a random subset of 60 putatively uninformative genes that are non-DE, non-DV, and non-DS with BY-adjusted *P*-value of 1.

Gene set functional enrichment analysis was done using WebGestalt.^57,58^ We explored enriched biological processes in the GO functional database using over representation analysis (ORA) of the following 5 gene lists: (i) high confidence DE genes obtained the intersection of sets of DE genes detected using SIEVEseq, edgeR, DESeq2, voom, tweeDEseq, ALDEx2, DSS and Wilcoxon; (ii) non-DE, DV and/or DS genes; (iii) pure DV genes; (iv) pure DS genes; and (v) the union of DE, DV, and DS genes. The human genome reference set was chosen. Only the top 20 GO terms were included, and affinity propagation was used for redundancy reduction. GO hierarchies were checked using WebGestalt's GOView application.^57^

To improve confidence in the reliability of biological processes inferred using the DE, DV and DS genes in the Mayo RNA-Seq dataset, we performed additional cross-methodological and cross-data validations. We applied Gene Set Enrichment Analysis^59^ (GSEA) on the Nakayama dataset to detect GO biological processes that are significantly enriched. Recovering a substantial proportion of significant GO biological processes across independent AD cohorts using GSEA provides cross-methodological support that the DE, DV and DS genes detected using SIEVEseq carry meaningful biological signals. To this end, we constructed a composite ranking statistic *U* that accentuates the most prominent distributional divergence of each gene in the Nakayama dataset. Specifically, we assigned each gene a composite score based on the difference of means ($\Delta \hat{\mu }$), the log_2_ fold-change of the standard deviation ratio $\left(\hat{\psi }\right)$, and the difference of skewness ($\Delta \hat{\gamma }$; see $\Delta \hat{\mu }$, $\hat{\psi }$, and $\Delta \hat{\gamma }$ in Materials Section S2). Thus, we defined $U=S\;\cdot \max\{|\Delta \hat{\mu }|,|\hat{\psi }|,|\Delta \hat{\gamma }|\}$, where *S* is the sign of the component with the largest magnitude. This statistic prioritizes genes exhibiting the largest shift in any of the 3 distributional moments, while preserving the direction of the effect.

### 2.7. Computing tools and environment

Computational work for DE, DV and DS tests was done using a MacBook Pro (macOS Monterey version 12.5) with 32 GB RAM, an M1 Pro chip and a 10-core CPU. The R (version 4.2.1)^60^ computing environment was used. Materials S6 gives the complete list of R packages used. For converting ENSEMBL gene ID to gene symbol, we used the application programming interface of BioTools.fr.^61^

## 3. Results

### 3.1. Parametric simulation study

**Fig. 1.**
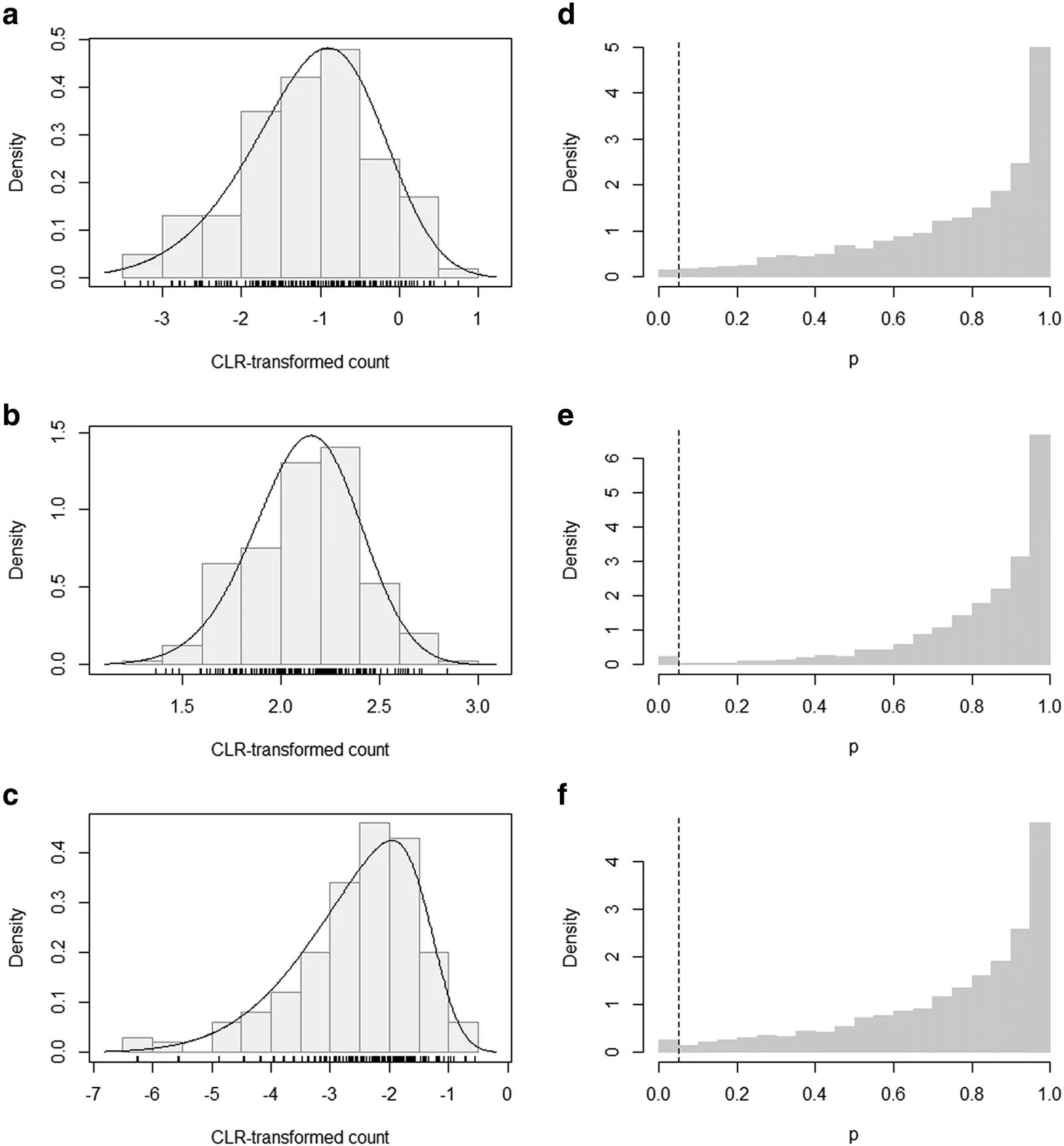
Histograms of CLR-transformed counts for 3 selected genes with fitted skew-normal curve for a) the Valentim dataset ($\hat{\mu }=-1.256\;(s.e.=0.061)$, $\hat{\sigma }=0.861\;(s.e.=0.046)$ and $\hat{\gamma }=-0.450\;(s.e.=0.172)$); b) the Kelmer dataset ($\hat{\mu }=2.119\;(s.e.=0.019)$, $\hat{\sigma }=0.274\;(s.e.=0.014)$ and $\hat{\gamma }=-0.232\;(s.e.=0.196)$); c) the Zhou dataset ($\hat{\mu }=-2.564\;(s.e.=0.073)$, $\hat{\sigma }=1.040\;(s.e.=0.057)$ and $\hat{\gamma }=-0.799\;(s.e.=0.071)$). Distribution of the *P*-values of the KS goodness-of-fit tests of the skew-normal model for genes in the simulated d) Valentim dataset, e) Kelmer dataset, and f) Zhou dataset. The skew-normal model gives good fit to about 99.2%, 98.9%, and 98.7% of the genes in d), e), and f), respectively. The dashed line corresponds to the threshold *P*-value of 0.05.

**Fig. 2.**
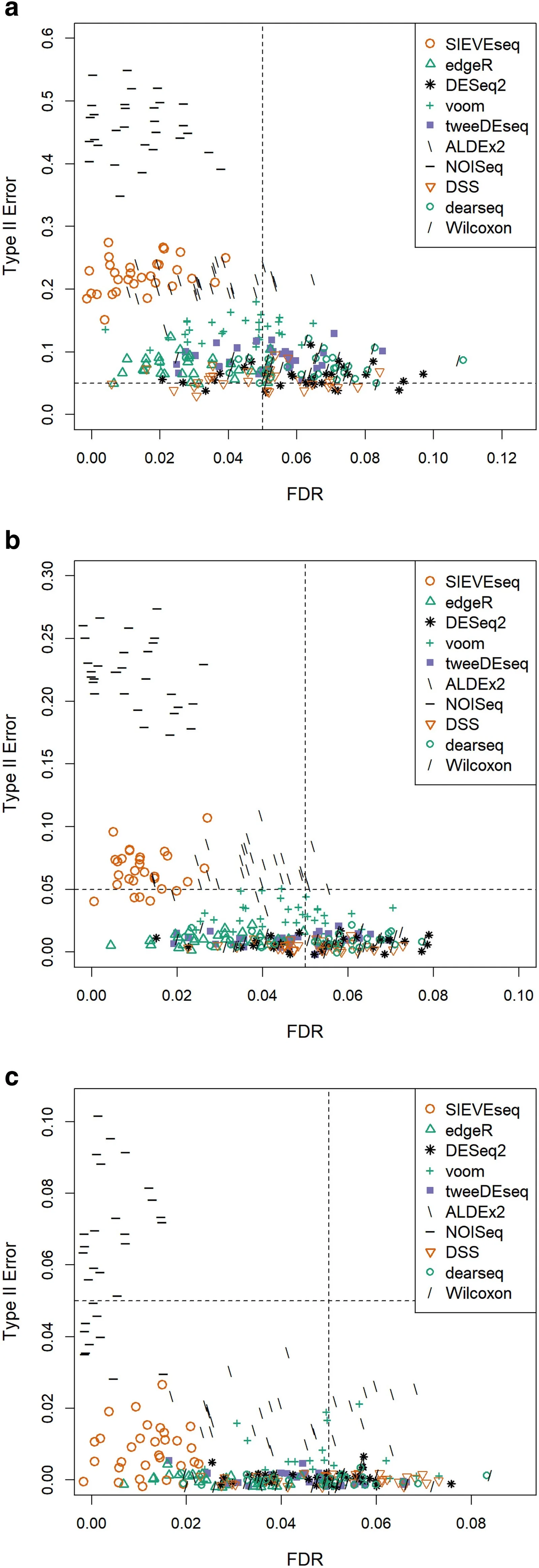
Scatter plots of the probability of Type II error (*β*) against FDR for simulated data from the Valentim dataset (30 instances) for 3 sample size per group scenarios: a) 30, b) 50, and c) 100. Dashed lines represent the desired threshold FDR and *β* of 0.05.

**Fig. 3.**
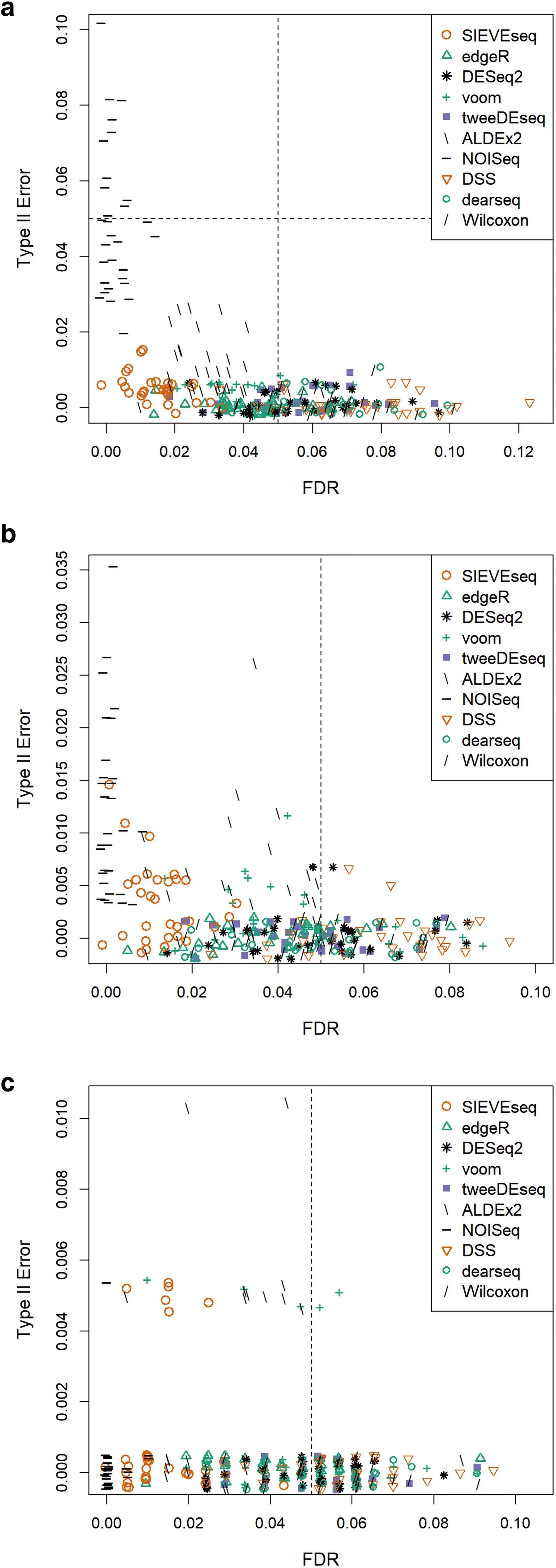
Scatter plots of the probability of Type II error (*β*) against FDR for simulated data from the Kelmer dataset (30 instances) for 3 sample size per group scenarios: a) 30, b) 50, and c) 100. Dashed lines represent the desired threshold FDR and *β* of 0.05.

**Fig. 4.**
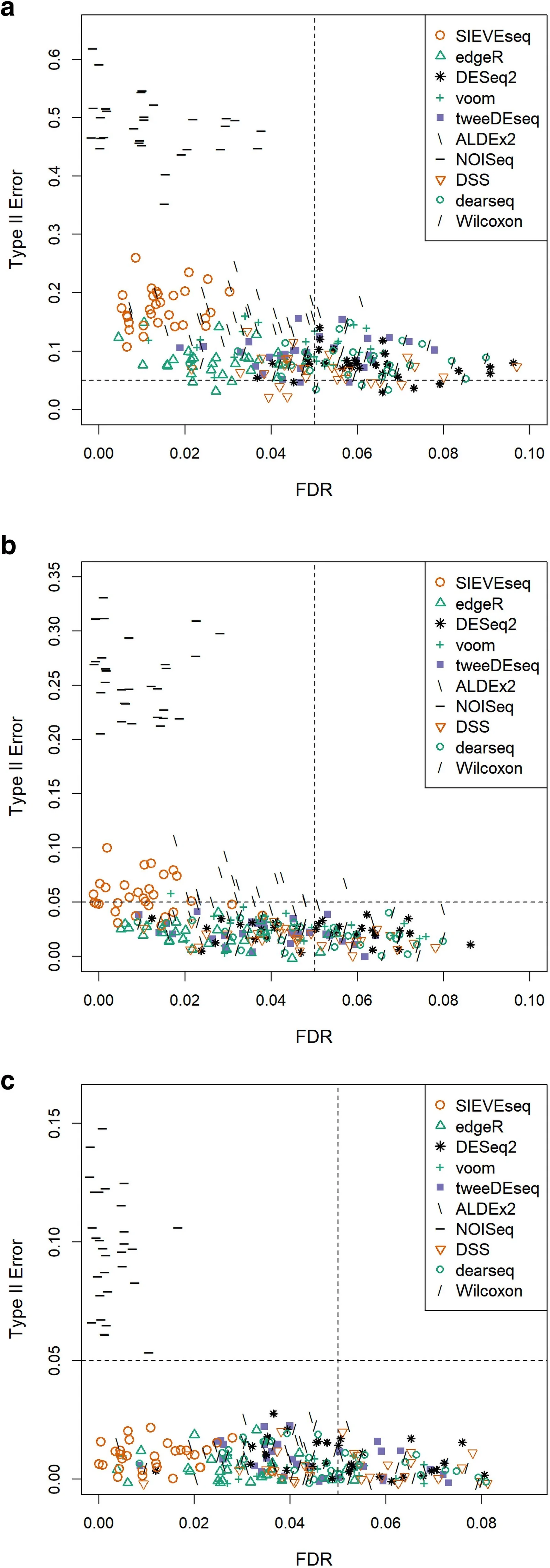
Scatter plots of the probability of Type II error (*β*) against FDR for simulated data from the Zhou dataset (30 instances) for 3 sample size per group scenarios: a) 30, b) 50, and c) 100. Dashed lines represent the desired threshold FDR and *β* of 0.05.

The skew-normal distribution fits the CLR-transformed count data of genes in the 3 datasets well, with 99.2%,98.9%, and 98.7% of genes having KS test *P*-values above 0.05 (Fig. 1). Graphical summaries of the parametric simulation results are given in Figs. 2 to 4. For brevity, details of the summary statistics of the performance metrics are given only for the Valentim dataset (Table 2). For the other 2 simulations, see Tables S1 and S2.

**Table 2.** The mean of FDR^a^, mean probability of Type II error (β^b^), and percentage of simulated instances with FDR <0.05 and *β* < 0.05 (*θ*^c^) for each of the 10 DE tests applied to data simulated from the Valentim dataset (30 instances) at 3 different sample sizes.

Method	Sample size per group		
	30	50	100
SIEVEseq	0.014 (0.011)	0.012 (0.006)	0.012 (0.007)^a^
	0.221 (0.028)	0.066 (0.015)	0.008 (0.007)^b^
	0%	13.3%	100%^c^
ALDEX2	0.034 (0.014)	0.038 (0.011)	0.041 (0.015)
	0.210 (0.022)	0.066 (0.016)	0.017 (0.008)
	0%	10%	66.7%
NOISeq	0.013 (0.011)	0.009 (0.008)	0.004 (0.005)
	0.458 (0.047)	0.223 (0.026)	0.062 (0.020)
	0%	0%	30%
edgeR	0.029 (0.013)	0.031 (0.012)	0.029 (0.010)
	0.076 (0.018)	0.010 (0.005)	0.000 (0.001)
	0%	96.7%	100%
DESeq2	0.062 (0.019)	0.054 (0.016)	0.047 (0.012)
	0.060 (0.017)	0.007 (0.005)	0.000 (0.002)
	6.7%	40%	50%
Voom	0.062 (0.019)	0.045 (0.012)	0.046 (0.014)
	0.134 (0.021)	0.031 (0.010)	0.004 (0.006)
	0%	60%	66.7%
DSS	0.049 (0.018)	0.051 (0.011)	0.051 (0.015)
	0.056 (0.015)	0.004 (0.004)	0.000 (0.000)
	13.3%	46.7%	46.7%
Dearseq	0.063 (0.016)	0.053 (0.015)	0.047 (0.014)
	0.077 (0.017)	0.009 (0.005)	0.000 (0.001)
	0%	33.3%	50%
Wilcoxon	0.047 (0.012)	0.045 (0.012)	0.045 (0.014)
	0.123 (0.024)	0.022 (0.009)	0.001 (0.003)
	0%	76.7%	73.3%
tweeDEseq	0.052 (0.014)	0.046 (0.014)	0.044 (0.012)
	0.089 (0.019)	0.010 (0.005)	0.000 (0.001)
	0%	56.7%	73.3%

Standard deviation in parentheses.

Overall, SIEVEseq, ALDEx2 and NOISeq prioritize FDR control over *β*. In all 3 simulations, these 3 methods, along with edgeR, are the top 4 methods with lower mean FDR. Specifically, NOISeq generally has the lowest mean FDR, followed by SIEVEseq, edgeR, and ALDEx2 in the simulated Valentim and Zhou datasets. In the simulated Kelmer dataset, NOISeq again has the lowest FDR, followed by SIEVEseq, ALDEx2, and edgeR. However, we note that NOISeq also has the highest mean *β* among all DE methods compared. ALDEx2 has relatively higher mean *β*, followed by SIEVEseq and edgeR. In all 3 simulations, edgeR has mean FDR that is relatively constant at about 0.03 (range: 0.026 to 0.042) across the 3 sample size scenarios. Furthermore, its mean *β* is at most about 0.08 at n=30 and almost 0 as *n* increases to 100. In contrast, DESeq2, voom, tweeDEseq, DSS, dearseq, and the Wilcoxon rank-sum test control FDR at around 0.05, with generally greater variation than edgeR's. All methods have decreasing *β* as sample size increases.

At n=100, only SIEVEseq has θ=100% for all 3 simulations (see Tables 2 and  S1 and S2). SIEVEseq, edgeR, and ALDEx2 have *θ* ranging from 66.7%to100%, 70%to100%, and 83.3%to100% for the 3 simulations, compared with 46.7%to73.3%, 26.7%to67.7%, and 43.3%to66.7% for DESeq2, voom, tweeDEseq, DSS, dearseq, and the Wilcoxon rank-sum test. NOISeq shows inconsistent performance, with *θ* ranging from 0% to 100%.

**Fig. 5.**
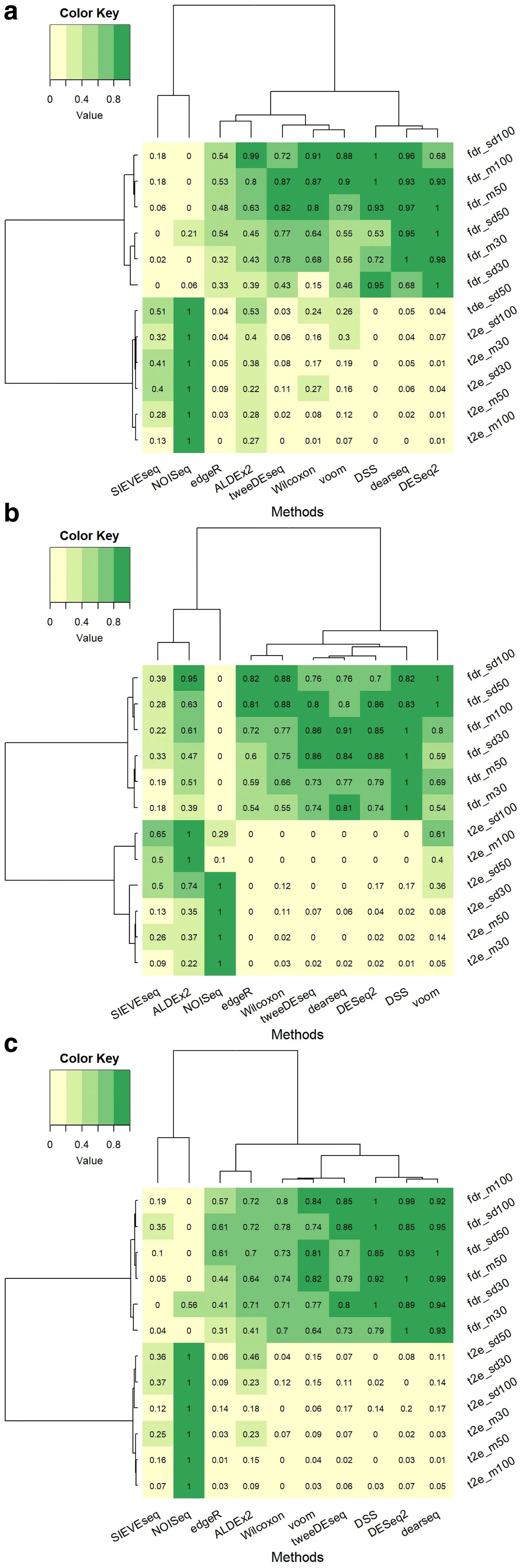
Cluster heatmaps showing similarity of DE methods (columns) with respect to summary statistics (rows) of FDR and probability of Type II error (*β*) at 3 sample size scenarios (*n* = 30, 50, 100) for the a) Valentim dataset, b) Kelmer dataset, and c) Zhou dataset. The number in a cell represents the mean (over 30 instances) of the summary statistic of a specific feature. fdr, FDR; m, mean; Sd, standard deviation; t2e, probability of Type II error; numerical suffixes indicate corresponding sample size scenario.

The cluster heatmap (Fig. 5) shows that NOISeq has mean *β* that is consistently the highest, in contrast with its mean FDR, which is consistently the lowest. SIEVEseq is similar to NOISeq with respect to mean FDR performance, but excels with substantially lower mean *β*. This suggests that SIEVEseq maintains the conservativeness of NOISeq without excessive compromise in *β*. Conversely, while other methods often have lower mean *β* values, their mean FDR values are correspondingly higher.

SIEVEseq took longer time to run for small sample sizes, but its speed improved for larger sample sizes (Table S3). At n=100 for all 3 simulations, edgeR, DESeq2, voom, DSS, dearseq, and the Wilcoxon rank-sum took about 5 s or less to complete, whereas NOISeq, ALDEx2 and SIEVEseq took about 11 to 46 s. The slowest method was tweeDEseq, which was 14 to 17 times slower than the second (ALDEx2) and the third (SIEVEseq) slowest methods, respectively.

The result of the sensitivity analysis for different choices of pseudo-values shows that 0.5 generally provides better control of both FDR and *β*, compared with the smaller pseudo-values of 0.01 and 0.1, for data simulated from the Valentim, Kelmer, and Zhou datasets across 3 sample size scenarios (Table S4; Figs. S2 to S4). Specifically, at sample size of 30, all 3 pseudo-values give very similar *β* values for all the simulated datasets. For moderately large sample size of 50, using 0.5 as the pseudo-value tends to provide uniformly better control of both FDR and *β*.

### 3.2. Nonparametric simulation study

**Fig. 6.**
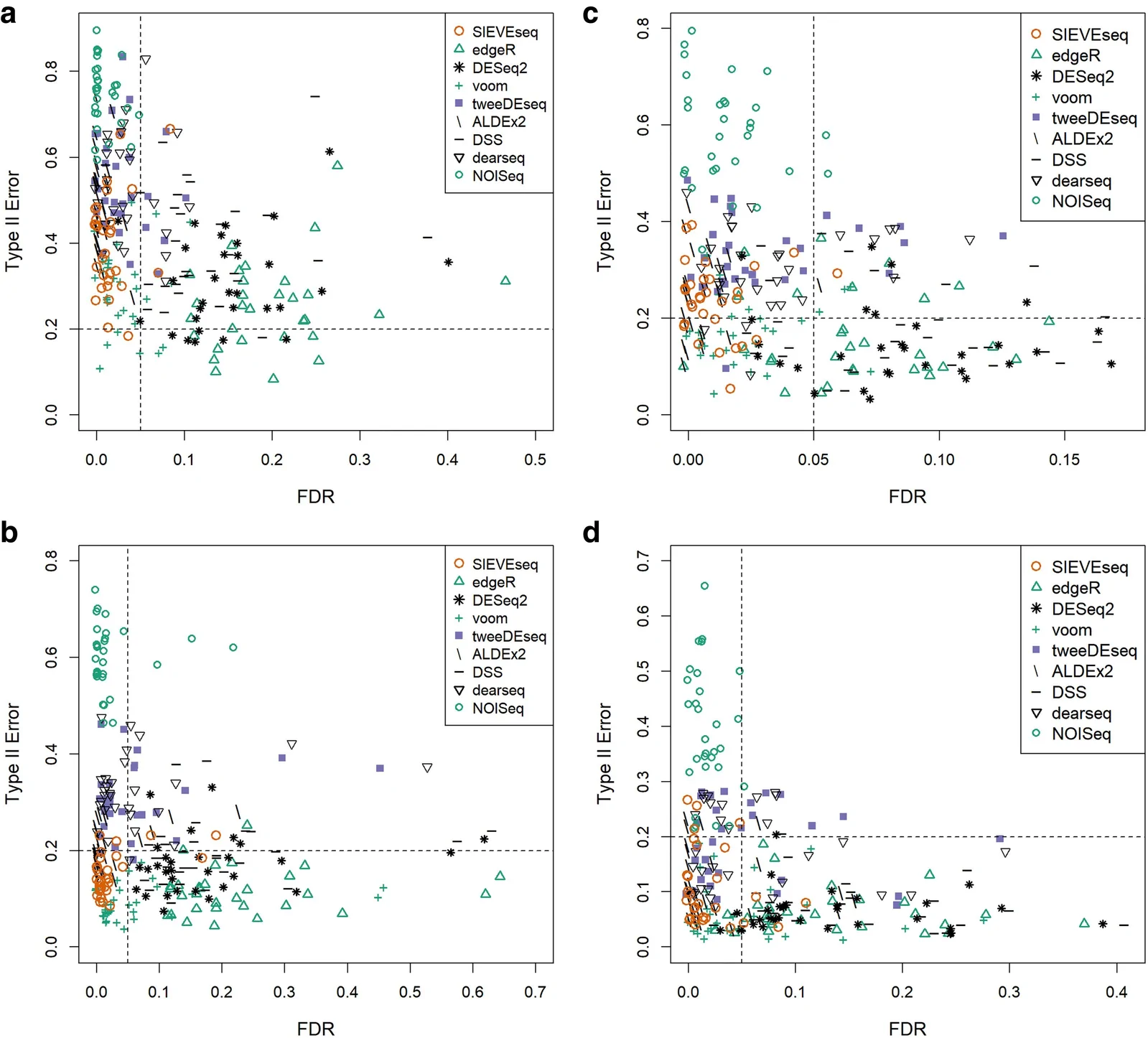
Scatter plots of the probability of Type II error (*β*) against FDR for simulated data from the a) Dolgalev dataset (30 instances) with a sample size of 30 per group; b) Dolgalev dataset (30 instances) with a sample size of 50 per group; c) Furusawa dataset (30 instances) with a sample size of 30 per group; and d) Furusawa dataset (30 instances) with a sample size of 50 per group. The vertical and horizontal dashed lines represent the target FDR threshold of 0.05 and the target *β* threshold of 0.2, respectively.

Graphical summaries of the nonparameteric simulation results are given in Fig. 6. For brevity, details of the summary statistics of the performance metrics for the Dolgalev and Furusawa datasets are given in Tables S5 and S6, respectively. In these scenarios, SIEVEseq, ALDEx2, and NOISeq demonstrated superior FDR control compared to conventional parametric methods. SIEVEseq maintained a low mean FDR across all conditions (range: 0.012 to 0.025). In contrast, widely used methods such as edgeR, DESeq2, and DSS failed to control the FDR within the nominal 0.05 level, with mean FDRs reaching as high as 0.231 for edgeR for the Dolgalev dataset simulation (n=50). NOISeq maintained the most stringent FDR control in some cases, but it consistently exhibited the highest *β* among all compared methods (eg β=0.749 at n=30 for the Dolgalev dataset), indicating low power in detecting true DE genes. SIEVEseq and ALDEx2 provided a better balance, with SIEVEseq consistently achieving lower *β* values than ALDEx2 across all scenarios.

SIEVEseq was the most reliable method with θ=83.3%, whereas for most other methods, including edgeR and DESeq2, θ dropped to 0%. Similarly, in the Furusawa dataset simulation with n=50, SIEVEseq achieved the highest *θ* of 76.7%. These results suggest that SIEVEseq is comparatively more robust against complexities of RNA-Seq data distributions with moderately large sample sizes by providing stable FDR control and maintaining competitive *β* under noncanonical models of count data distribution.

**Fig. 7.**
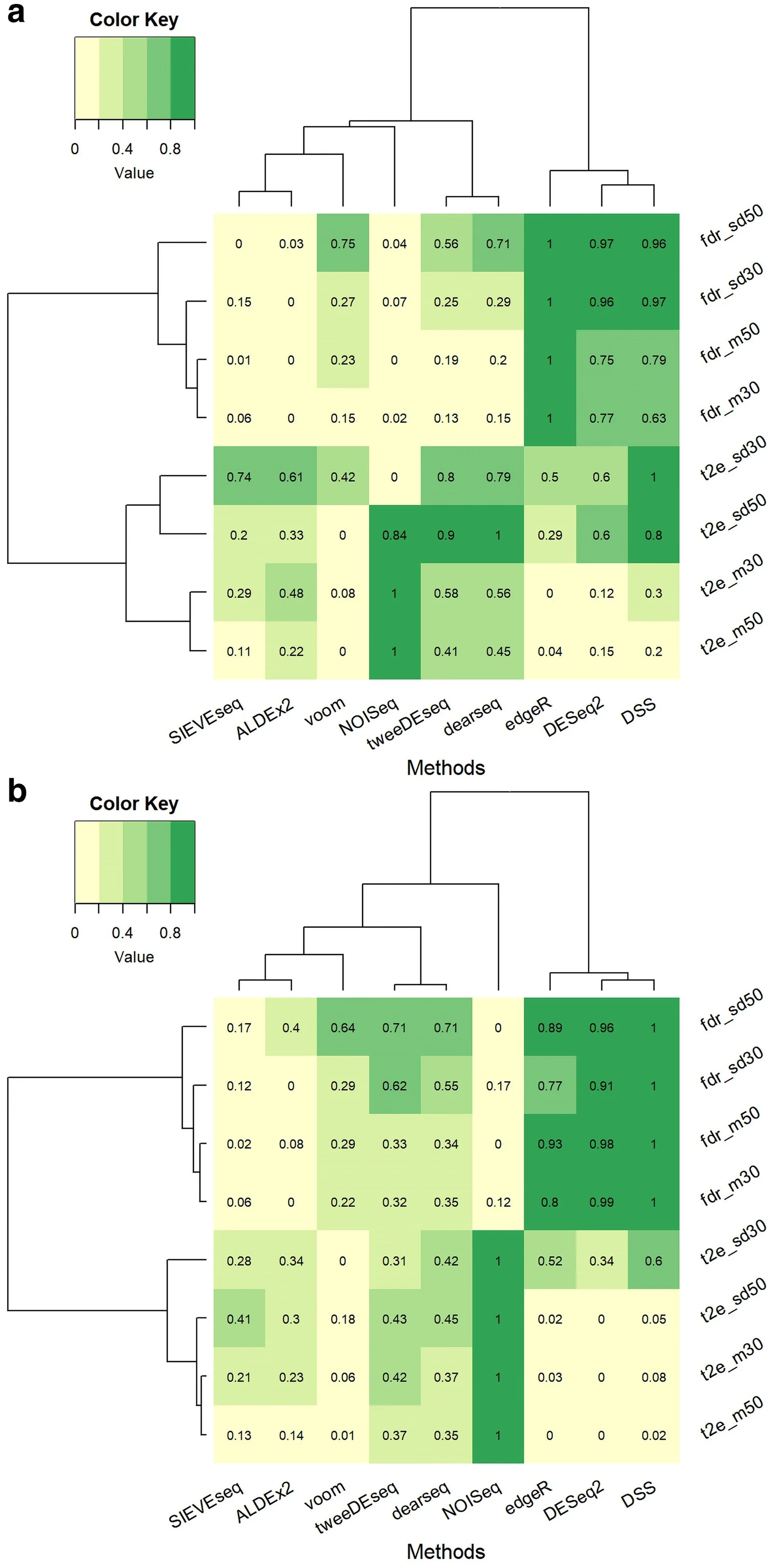
Cluster heatmaps showing similarity of DE methods (columns) with respect to summary statistics (rows) of FDR and probability of Type II error (*β*) at 2 sample size scenarios (*n* = 30, 50) for the a) Dolgalev dataset and b) Furusawa dataset. The number in a cell represents the mean (over 30 instances) of the summary statistic of a specific feature. fdr, FDR; m, mean; sd, standard deviation; t2e, probability of Type II error; numerical suffixes indicate corresponding sample size scenario.

The cluster heatmaps (Fig. 7) show that edgeR, DESeq2, and DSS cluster closely together. These 3 methods had the highest mean and standard deviation of FDR, which indicate a failure to control false discoveries. In contrast, SIEVEseq and ALDEx2 had consistently similar low mean FDR levels. NOISeq achieved the lowest mean FDR, but its *β* remained the highest across both datasets. Interestingly, SIEVEseq matched the low FDR performance of NOISeq but excelled with substantially lower mean *β*. Thus, it seems that SIEVEseq successfully maintains the conservativeness of NOISeq without an excessive increase in *β*, even in a nonparametric simulation scenario.

### 3.3. Analysis of the Mayo RNA-Seq dataset

Most of the genes in both control (98.5%; 18385/18664) and AD groups (99.5%; 18571/18664) have CLR-transformed count data that are well-fitted (*P*-value >0.05) by the skew-normal distribution (Fig. S5). We performed DE analysis of the Mayo RNA-Seq dataset by comparing SIEVEseq against edgeR, DESeq2, voom, tweeDEseq, ALDEx2, DSS, Wilcoxon, and NOISeq. We excluded dearseq because it is unable to provide output for the mean expression levels, rendering the significance score method used inapplicable. Figure S7 shows the number of DE genes unique to a DE method or common to multiple DE methods (for complete list, see Table S13). DSS detected the most DE genes (4171), followed by DESeq2 (4155), edgeR (3942), voom (3636), tweeDEseq (3553), Wilcoxon (3330), ALDEx2 (3230), SIEVEseq (2773), and NOISeq (2014). That NOISeq detected the least number of DE genes is consistent with findings from the simulation studies that show its poor control of probability of Type II error. Table S14 shows the list of DE, DV (2276) and DS (1024) genes detected using SIEVEseq (see also Fig. S6). With respect to computational time, SIEVEseq completed the DE, DV, and DS tests concurrently in approximately 5.5 min. The run time for other methods (in increasing order) is as follows: 20 s for DSS; about 30s for Wilcoxon; about 45s for edgeR, DESeq2, limma-voom and NOISeq; about 40 min for ALDEx2; and about 2.5 h for tweeDEseq.

We defined the intersection of DE genes detected using each method as the high confidence gene set. Here, we excluded NOISeq because simulation studies suggested that it has a high false negative rate. Comparing SIEVEseq to 3 popular DE tests (ie edgeR, DESeq2, and voom), 98.7%(2736/2773)) of DE genes identified by SIEVEseq are also detected by edgeR, DESeq2, or voom; 91.6%(2540/2773) are detected by edgeR, DESeq2, and voom; only 1.3%(37/2773) are uniquely detected by SIEVEseq. Furthermore, 93.1%(2583/2773), 94.3%(2615/2773), and 97.0%(2691/2773) of DE genes detected by SIEVEseq are also identified by edgeR, DESeq2 and voom, respectively. In comparison with ALDEx2, tweeDEseq, DSS and Wilcoxon, about 94.8%(2630/2733), 91.1%(2527/2773), 94.6%(2622/2773), and 91.2%  (2528/2773) of DE genes identified by SIEVEseq are also detected by these 4 methods, respectively. Overall, 84.9%  (2354/2773) of DE genes detected by SIEVEseq are also detected by all the other 7 DE methods.

Table 3 gives the distribution of gene classes returned by SIEVEseq. The majority of genes are neither DE, DV, nor DS (∼70%; 13030/18664). For the remaining 30% of genes, focusing solely on DE genes means that only about 49% (2773/5634) of them would be considered in downstream analyses. The ability of SIEVEseq to detect the remaining 51% (2861/5634) of DV and/or DS genes that cannot be detected using DE methods sets it apart from all current methods. See Figs. S8 to S12 for examples of distributional variation.

**Table 3.** Eight classes of genes identified by SIEVEseq for the Mayo RNA-Seq dataset.

Gene class	000	100	010	001	110	101	011	111
Number of genes	13,030	2,284	1,843	919	303	155	99	31

000, non-DE, non-DV, and non-DS; 100, DE, non-DV, and non-DS; 010, non-DE, DV, and non-DS; 001, non-DE, non-DV, and DS; 110, DE, DV, and non-DS; 101, DE, non-DV, and DS; 011, non-DE, DV, and DS; 111, DE, DV, and DS.

Table S7 summarizes the sets of pure DV, pure DS, and DVS (non-DE, DV, and DS) genes detected using SIEVEseq that intersect with the DE gene sets identified by edgeR, DESeq2, voom, ALDEx2, tweeDEseq, DSS, and Wilcoxon. The key observation is that only 497/2861=17.4% of the genes in the union set of the pure DV, pure DS and DEV (DE, DV, and non-DS) genes can be detected collaterally by the 7 DE methods considered. This suggests that SIEVEseq detects a large number of genes with significant variability and skewness in gene expression distribution that standard DE methods fail to identify.

**Fig. 8.**
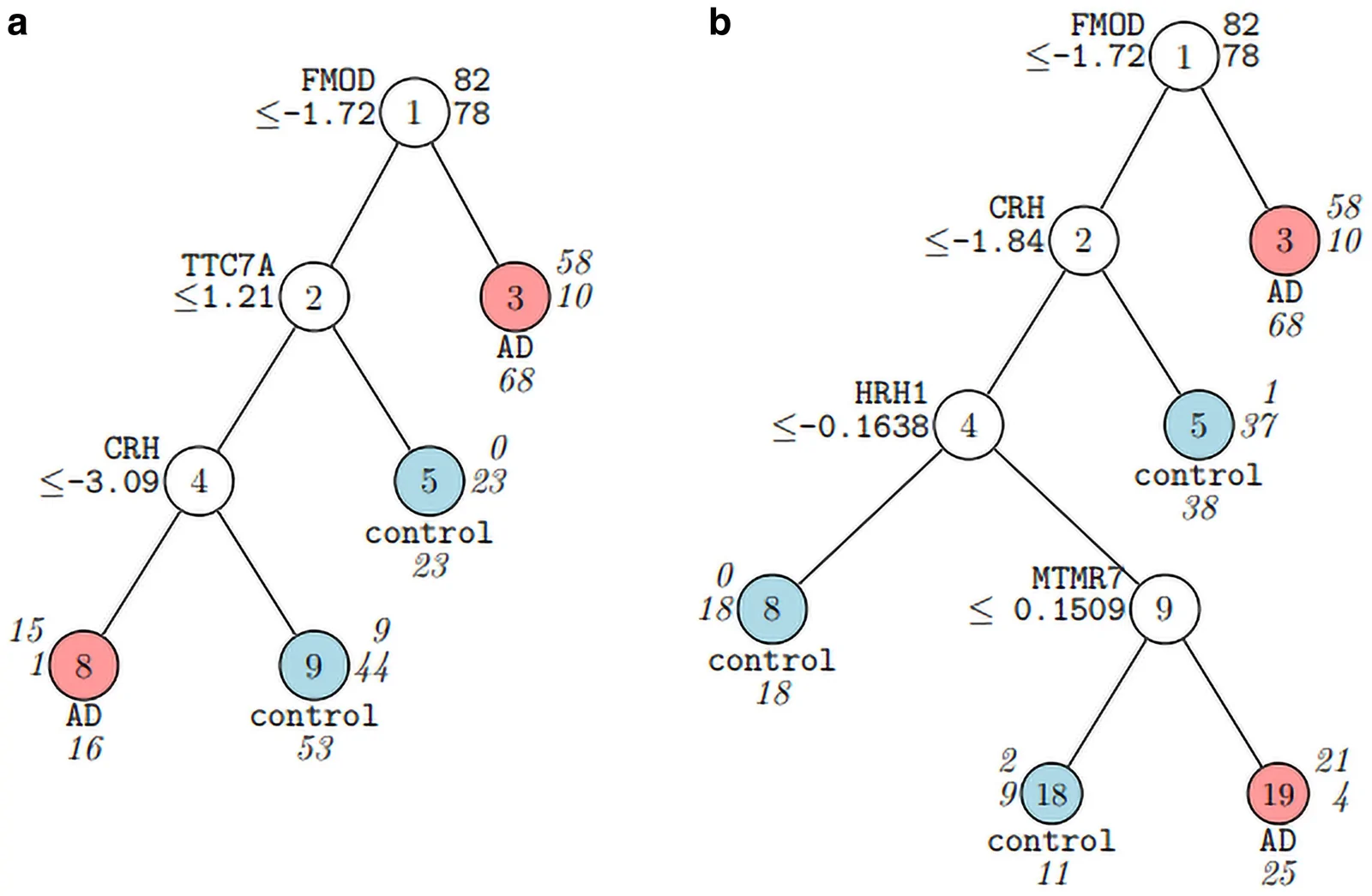
GUIDE v.40.3 0.250-SE classification tree for predicting using equal priors and unit misclassification costs. At each split, an observation goes to the left branch if and only if the condition is satisfied. Predicted classes and sample sizes (in *italics*) are printed below terminal nodes; class sample sizes for= and beside nodes. a) Classification tree with *TTC7A*; b) classification tree without *TTC7A*. *FMOD*: DE gene; *TTC7A*: DS gene; *CRH*: DE gene; *HRH1*: DV gene; *MTMR7*: DS gene.

Figure 6 shows that DE, DV, and DS genes are represented among 2 alternative GUIDE trees for discriminating AD from the control state. The fibromodulin gene (FMOD; DE gene) is critical for enabling the identification of 70.7%(58/82) AD cases. A DS gene—tetratricopeptide repeat domain 7A (*TTC7A*)—is crucial for enabling the prediction of the remaining AD cases (18.3%;15/82) using the corticotropin releasing hormone gene (Fig. 8a). If *TTC7A* is removed, the resulting tree uses another DS gene—myotubularin related protein 7 gene (*MTMR7*) to predict the remaining 25.6%(21/82) AD cases (Fig. 8b). Overall, the first tree has estimated accuracy, sensitivity, and specificity of 87.5%(140/160), 89.0%(73/82) and 85.9%(67/78), respectively. For second tree, the estimated accuracy, sensitivity, and specificity is 89.4%(143/160), 96.3%(79/82), and 82.1%(64/78), respectively. See Materials S5 for a brief summary of the biological relevance of genes in both trees.

The estimated generalization error of GUIDE was approximately the same (∼17%) regardless of whether DE, DV and DS gene sets were used separately, or collectively. In contrast, the estimated generalization error of GUIDE using genes that are neither DE, DV nor DS genes was more than twice as large (39.4%; 63/160). This finding suggests that the gene sets detected using SIEVEseq are informative.

**Fig. 9.**
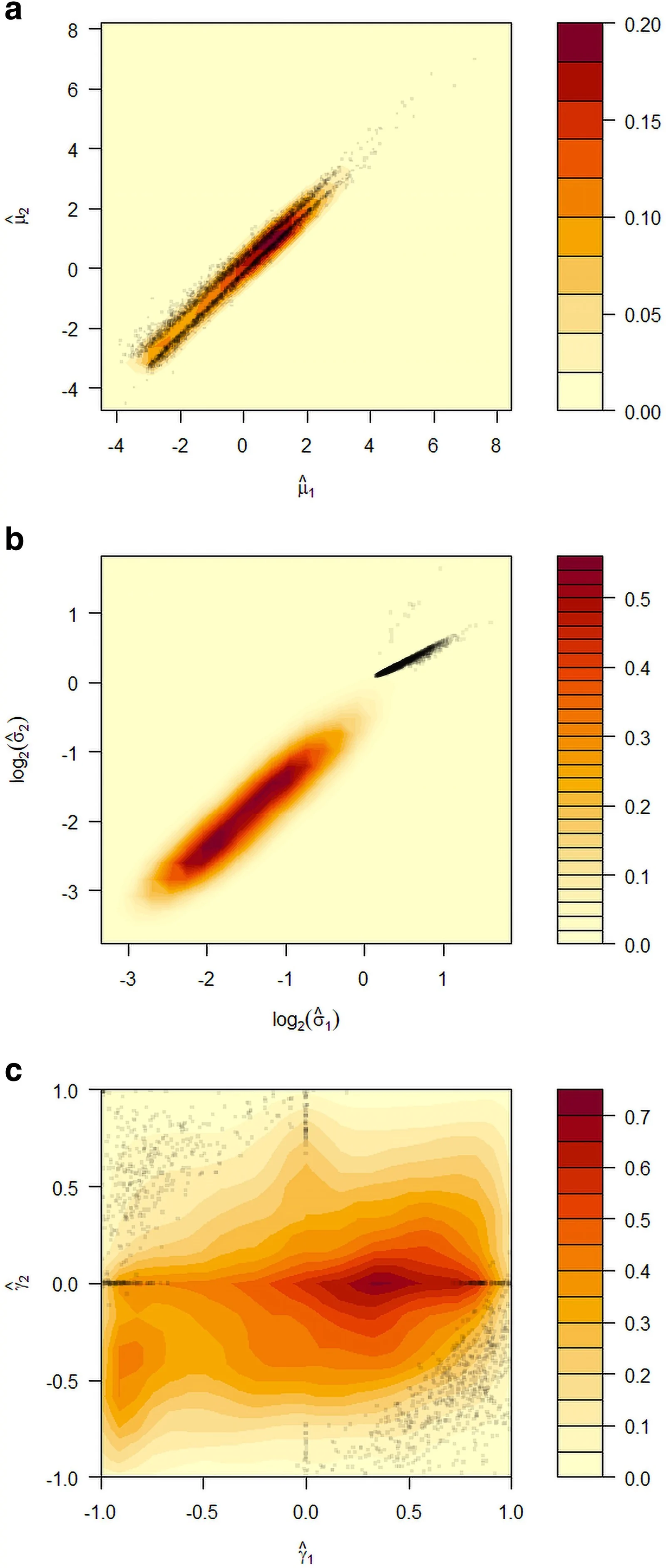
Contour plots of a) ${\hat{\mu }}_{1}$ vs. ${\hat{\mu }}_{2}$, b) $\log_{2}\;\;{\hat{\sigma }}_{1}$ vs. $\log_{2}\;\;{\hat{\sigma }}_{2}$, and (c) ${\hat{\gamma }}_{1}$ vs. ${\hat{\gamma }}_{2}$, with density colour keys on the right. The Subscripts 1 and 2 denote the control group and AD group, respectively. Significant genes are indicated as black squares. Range of the estimated parameters: ${\hat{\mu }}_{1}\in (-4.495,\;\;8.457)$, $\log_{2}\;{\hat{\sigma }}_{1}\in (-3.344,\;\;1.867)$, ${\hat{\gamma }}_{1}\in (-0.994,\;\;0.994)$, ${\hat{\mu }}_{2}\in (-4.763,\;\;8.190)$, $\log_{2}\;{\hat{\sigma }}_{2}\in (-3.769,\;\;1.817)$, ${\hat{\gamma }}_{2}\in (-0.993,\;\;0.994)$.

The joint distribution of ${\hat{\mu }}_{1}$ and ${\hat{\mu }}_{2}$ appears approximately bivariate normal (Fig. 9a), with DE genes distributed on the periphery of the outermost probability ellipse. Similarly, the joint distribution of $\log_{2}\;{\hat{\sigma }}_{1}$ and $\log_{2}\;{\hat{\sigma }}_{2}$ is also approximately bivariate normal (Fig. 9b). Most genes have $\hat{\sigma }$ values between 0 and 1 in the control and the AD group, and are non-DV; DV genes generally have $\hat{\sigma }$ >1, with most of them having smaller $\hat{\sigma }$ in the AD group compared to the control group (see also Li and Khang^30^). Finally, the joint distribution of ${\hat{\gamma }}_{1}$ and ${\hat{\gamma }}_{2}$ appears to be bimodal (Fig. 9c), with DS genes distributed towards the upper left and bottom right quadrants. In the AD group, genes tend to have $\hat{\gamma }$ close to 0; thus, we may expect to see more genes with Gaussian-distributed CLR-transformed expression values. For genes in the control group, $\hat{\gamma }$ tends to be relatively more uniformly distributed; thus, distributions of CLR-transformed expression values are generally left or right-skewed.

### 3.4. Functional enrichment analysis

Table 4 presents concise groupings of 18 GO terms obtained using all 5 gene lists into 7 biological aspects. For details, see Tables S8 to S12. The results indicate that an enrichment analysis that considers only conventional DE genes detects cell adhesion, extracellular structure organization, blood vessel development, and wound healing as enriched biological processes. Interestingly, consideration of non-DE but DV and/or DS genes reveals involvement of membrane protein localizations in AD pathogenesis, which is well-known, while simultaneously uncovering RNA catabolism. While the 4 enriched biological processes that are associated with pure DS gene list have FDR of about 0.61, they remain biologically interesting as studies of their involvement in AD have been reported. Importantly, enrichment analysis using the list of DE, DV, and DS genes from SIEVEseq not only recovers many hierarchically-related processes detected using just DE genes, or just DV and/or DS genes, but also uniquely detects the positive regulation of cell communication process. To summarize, our findings suggest that incorporating DV and DS analyses alongside DE analysis enables a more comprehensive understanding of important biological processes involved in a disease.

**Table 4.** Functional enrichment in GO biological processes for the DE, DV, and DS genes for the control vs. AD comparison.

Biological aspects	GO ID	GO description	Gene list (size)				
			A	B	C	D	E
Cellular protein localization	GO:0034613	Cellular protein localization [+4]			✓		
	GO:0072599	Establishment of protein localization to ER [+2]	✓				
	GO:0090150	Establishment of protein localization			✓	✓	
		to membrane [+3]					
	GO:0045047	Protein targeting to ER [+1]				✓	
	GO:0006614	SRP-dependent cotranslational protein			✓		
		targeting to membrane [0]					
Cell communication	GO:0022610	Biological adhesion [+2]	✓				
	GO:0031589	Cell-substrate adhesion [0]	✓	✓			
	GO:0010647	Positive regulation of cell communication		✓			
	GO:0070848	Response to growth factor					
Matrix biology	GO:0030198	ECM organization	✓				
Angiogenesis	GO:0001568	Blood vessel development	✓	✓			
	GO:0042060	Wound healing		✓			
Regulation of gene expression	GO:0006412	Translation			✓	✓	
	GO:0006401	RNA catabolic process				✓	
Immune response	GO:0019884	Antigen processing and presentation of exogenous antigen					✓
	GO:0045619	Regulation of lymphocyte differentiation					✓
Oxidative stress	GO:0019363	Pyridine nucleotide biosynthetic process					✓
	GO:0019752	Carboxylic acid metabolic process					✓

The size of a gene list refers to the number of gene symbols in the list that are unambiguously mapped to unique Entrez gene IDs. For hierarchical GO terms, the reference GO term is indicated by [0]; parent/child terms have a +/− sign, with the numerical suffix indicating the hierarchy levels above/below the reference GO term. The FDRs of GO terms returned using all gene lists except that of pure DS have order of magnitude <$10^{-9}$. For the latter gene list, the FDRs of the GO terms are all about 0.61. A: DE, DV, DS genes (4,907); B: high confidence DE genes (1,981); C: non-DE, DV, DS genes (2,497); D: pure DV genes (1,578); and E: pure DS genes (811).

A search of the AD literature reveals that many of these aspects in Table 4 have been investigated. Here, we briefly examine the literature that report on the association of these biological aspects with AD pathophysiology.

(i) Cellular protein localization

The endoplasmic reticulum (ER) is a critical organelle for the biosynthesis, folding, modification and assembly of proteins. Under stress, protein biosynthesis demand may exceed protein-folding capacity at the ER lumen, thus resulting in the production of proteins that are partially folded, misfolded, or unfolded, which leads to a condition known as ER stress.^62^ Amyloid beta (Aβ) deposition in the ER is thought to produce ER stress, and excessive levels of such stress can trigger maladaptive unfolded protein response activation that produces excessive autophagy and induction of cell death pathways.^63^ Loss of the signal recognition particle (SRP) results in the mistargeting of genes bound for ER to the mitochondria, which damages mitochondrial structure.^64^

(ii) Matrix biology

Constituents of the extracellular matrix (ECM) such as chondroitin sulphate proteoglycan and perineuronal net are potentially neuroprotective against tau lesions in AD pathogenesis.^65^ In particular, deglycosylation of perineuronal net has only very recently been shown to be associated with tauopathy-induced gliosis and neurodegeneration in mouse models.^66^

(iii) Cell communication

Cell adhesion molecules have been shown to be linked to Aβ metabolism as well as involved in neuroinflammatory responses and vascular changes.^67^ Dysfunctional components of Wnt signalling have been shown to increase neuronal susceptibility to Aβ toxicity.^68^ Enhancing Wnt signalling may potentially mitigate synaptic pathology in AD.^69^

(iv) Angiogenesis

The involvement of angiogenesis in AD pathophysiology was hypothesized as early as the early 2000s^70^ and experimental support had accumulated since then.^71^ A fraction of AD cases may be caused by defects in brain wound healing.^72^ In a mouse model, overexpression of tau proteins induced changes in brain blood vessels that altered blood flow and led to cortical atrophy.^73^ Major findings implicating vascular channel dysfunction^74^ and pericyte-associated dysregulation of blood flow in brains of AD patients were reported in recent years.^75^

(v) Regulation of gene expression

The translation of the mRNA of the amyloid precursor protein is inhibited by the fragile X mental retardation protein (FMRP).^76,77^ Additionally, the reduced expression of an FMRP-binding protein known as the cytoplasmic FMRP-interacting protein 2 leads to accumulation of phosphorylated tau proteins and Aβ peptides.^78^ Meier et al. showed that tau-ribosome interaction in the ER impairs global RNA translation as well as the synthesis of an important synaptic protein that leads to synaptic dysfunction.^79^

(vi) Immune response

Neuroinflammation, whereby misfolded and aggregated proteins bind to surface receptors on microglia and astroglia to trigger innate immune response involving release of inflammatory mediators, is recognized as one of the hallmarks of AD pathophysiology.^80–82^ The effect of pathological differentiation of T cells during inflammation and the importance of antigen presentation through MHCII-positive microglia in AD pathophysiology were recently reviewed.^83,84^

(vii) Oxidative stress

The mitochondria, which is critical for cellular energy metabolism and redox homeostasis, is a major target of oxidative damage. Indeed, oxidative stress brought about by reactive oxygen species eventually leads to a runaway spiral towards cellular degeneration in AD.^85,86^ Pesini et al.^87^ hypothesized that the pathogenesis of late-onset AD in a subgroup of AD patients may be related to a decrease in the de novo synthesis of pyrimidine nucleotides, which leads to dysfunction of the oxidative phosphorylation system. Carboxylic acid metabolism is related to lipid metabolism, and lipid dyshomeostasis has been shown to be present during early stages of AD brains.^88^

### 3.5. Cross-methodology and cross-data validations

Out of the 18 benchmark GO terms identified from the Mayo RNA-Seq dataset using ORA, 16 (∼89%) were successfully evaluated within the GSEA framework (Table 5). We found substantial functional consistency: about 37.5% (6/16) showed significant enrichment in GSEA, with adjusted *P*-value <0.05. Relaxing the latter's threshold to 0.20, about 56% (9/16) of the GO terms were significantly enriched. These substantial overlap percentages suggest that the identified biological processes reflect genuine biological signals and appear stable across different datasets. Such consistency confirms that the detected DE, DV, and DS signals are likely robust and do not depend on a particular enrichment framework or a specific AD cohort. Thus, we conclude that inclusion of DV and DS genes enables the detection of signalling surveillance processes that govern heterogeneity of gene expression in cells, which is not possible with the use of DE genes only.

**Table 5.** Cross-methodology and cross-data validations of ORA-derived GO terms.

GO ID	Description	NES	Adjusted *P*-value
GO:0010647	Positive regulation of cell communication	1.49	0.0001^a^
GO:0045047	Protein targeting to ER	−1.85	0.0176^a^
GO:0019884	Antigen processing and presentation of exogenous antigen	1.69	0.0268^a^
GO:0070848	Response to growth factor	1.30	0.0268^a^
GO:0072599	Establishment of protein localization to ER	−1.69	0.0268^a^
GO:0045619	Regulation of lymphocyte differentiation	1.40	0.0358^a^
GO:0006614	SRP-dependent cotranslational protein targeting to membrane	−1.56	0.0891^b^
GO:0006401	RNA catabolic process	1.21	0.1190^b^
GO:0042060	Wound healing	1.21	0.1190^b^
GO:0006412	Translation	−1.11	0.2766
GO:0090150	Establishment of protein localization to membrane	−1.13	0.2772
GO:0001568	Blood vessel development	1.00	0.5868
GO:0019752	Carboxylic acid metabolic process	−0.99	0.5868
GO:0031589	Cell-substrate adhesion	0.98	0.5868
GO:0019363	Pyridine nucleotide biosynthetic process	0.76	0.8491
GO:0030198	ECM organization	−0.64	0.9958

Benchmark GO terms identified in the Mayo dataset were validated using GSEA in the independent Nakayama dataset. Enrichment was performed using a composite ranking score integrating DE, DV, and DS signals.

Adjusted *P*-value: ^a^*P* < 0.05 and ^b^*P* < 0.20.

NES, normalized enrichment score.

## 4. Discussion

In the Mayo RNA-Seq dataset, almost 50%(2773/5634) of genes that have significant difference in at least one aspect of the gene expression distribution can be detected using DE test. This affirms the value of conducting DE tests as a routine step in RNA-Seq data analysis. Importantly, DE tests cannot detect pure DV, pure DS, or DVS genes that constitute the remaining 50%(2861/5634) genes. As complex diseases are multifaceted, genomic studies that use DE genes for functional enrichment analysis would be disadvantaged by a narrow perspective. Here, querying the GO database using the gene lists generated by SIEVEseq substantially expands the landscape of biological processes that are potentially implicated in the pathogenesis of a disease, thus enabling their further exploration and investigation.

SIEVEseq may not be suitable for RNA-Seq studies with small sample sizes, since it generally requires larger sample sizes (50 or more per group) for reliable estimation of the standard deviation and skewness parameters. As the fit of the skew-normal model to CLR-transformed data is important, the result of testing the goodness-of-fit of the skew-normal model should be reported. Our present analysis was necessarily restricted to the Mayo RNA-Seq dataset, owing to the need for depth of analysis. We encourage more future analyses using SIEVEseq to ascertain whether the skew-normal distribution is indeed a ubiquitous feature of CLR-transformed RNA-Seq data.

To summarize, SIEVEseq unlocks the potential of RNA-Seq data by allowing a richer class of genes to be characterized with respect to their mean, standard deviation, and/or skewness characteristics. With the availability of SIEVEseq, substantial published RNA-Seq datasets of complex diseases may be fruitfully reanalysed to generate new hypotheses concerning involvement of hitherto unconsidered biological processes. Another possible application is the annotation of genes in gene-gene interaction networks using the gene classes generated by SIEVEseq, which can potentially facilitate interpretation through integration of domain knowledge. Last but not least, given that pseudo-bulk profiles retain biological variability and skewness at the individual, cell-type, or group levels, SIEVEseq's unique capacity to detect changes in these higher moments makes it a valuable tool for revealing important biological signals that are missed by conventional DE methods. This opens new opportunities for the reanalysis of large-scale pseudo-bulk scRNA-Seq datasets.

## Supplementary Material

dsag012_Supplementary_Data

## Data Availability

The SIEVEseq R package is available in CRAN at https://cran.r-project.org/web/packages/SIEVEseq. The source code and development version are available at https://github.com/Divo-Lee/SIEVEseq.
